# The *AtHSP17.4C1* Gene Expression Is Mediated by Diverse Signals that Link Biotic and Abiotic Stress Factors with ROS and Can Be a Useful Molecular Marker for Oxidative Stress

**DOI:** 10.3390/ijms20133201

**Published:** 2019-06-29

**Authors:** Nasser Sewelam, Kemal Kazan, Meike Hüdig, Veronica G. Maurino, Peer M. Schenk

**Affiliations:** 1 Plant Molecular Physiology and Biotechnology Group, Institute of Developmental and Molecular Biology of Plants, Heinrich Heine University, and Cluster of Excellence on Plant Sciences (CEPLAS), Düsseldorf 40225, Germany; 2 Botany Department, Faculty of Science, Tanta University, Tanta 31527, Egypt; 3 Commonwealth Scientific and Industrial Research Organisation (CSIRO) Agriculture, Queensland Bioscience Precinct, St Lucia, Queensland 4067, Australia; 4 Queensland Alliance for Agriculture and Food Innovation, University of Queensland, St Lucia, Queensland 4072, Australia; 5 Plant-Microbe Interactions Laboratory, School of Agriculture and Food Sciences, The University of Queensland, Brisbane, Queensland 4072, Australia

**Keywords:** Reactive oxygen species, small heat shock proteins, abiotic stress, biotic stress, abscisic acid, salicylic acid

## Abstract

Reactive oxygen species (ROS) are highly controlled signaling species that are involved in regulating gene expression in response to different environmental cues. The production of heat shock proteins (HSPs) is a key strategy that plants use to defend themselves against diverse stresses, including oxidative stress. In this study, expression patterns of the Arabidopsis *HSP17.4CI* gene, a cytosolic class I small HSP, were systematically profiled under different abiotic, biotic and oxidative stresses. Our data show that *HSP17.4CI* was early and highly induced by heat, cold, salt, drought and high-light. *HSP17.4CI* also showed high expression levels in Arabidopsis plants infected with the biotrophic pathogen *Pseudomonas syringae*, but not in response to the necrotrophic pathogens *Alternaria brassicicola* and *Fusarium oxysporum*. Oxidative stress treatments including H_2_O_2_ and the herbicide methyl viologen led to induction of *HSP17.4CI*. The plant hormones abscisic acid (ABA) and salicylic acid (SA) induced the expression of *HSP17.4CI*, whereas methyl jasmonate (MJ) did not affect the expression level of this gene. Furthermore, we found enhanced expression of *HSP17.4CI* in catalase mutant plants, which are deficient in catalase 2 activity and accumulate intracellular H_2_O_2_. Taken together, data presented here suggest that *HSP17.4CI* expression is regulated by various signals that connect biotic and abiotic stresses with ROS and can be used as a molecular marker for oxidative stress.

## 1. Introduction

Plants are continuously subjected to a broad range of biotic and abiotic stresses that negatively affect plant productivity. Therefore, revealing the relevant mechanisms of plant responses to stress is of great importance. Available evidence suggests that reactive oxygen species (ROS) production in plants is a common response to almost all environmental challenges [1,2,3]. ROS work as highly controlled signaling species that are able to deliver different environmental cues to the plant cell transcriptional machinery [4,5,6,7,8,9]. This signaling function of ROS is becoming more operative through their interactions with other various signaling components including phytohormones, G proteins, calcium ions and mitogen-activated protein kinases (MAPKs) [7]. Furthermore, ROS are reported to mediate cellular signals through direct interaction with transcription factors, including heat shock factors (HSFs). For example, HSFA2 and HSFA4A were reported to be induced by oxidative stress and involved in H_2_O_2_ sensing [10,11].

H_2_O_2_, the most widespread cellular ROS, is an uncharged molecule that defuses readily through biological membranes [12,13] and thus can efficiently deliver intracellular systemic signals [7]. Indeed, H_2_O_2_ has the ability to modulate the expressional behavior of many genes in various organisms. The expression of about one-third of *Saccharomyces cerevisiae* genes was shown to be modulated upon exposure to H_2_O_2_ [14]. In Arabidopsis, 175 out of 11,000 non-redundant expressed sequence tags were found to be regulated by H_2_O_2_ treatment [15]. Similarly, production of H_2_O_2_ in chloroplasts of Arabidopsis plants treated with methyl viologen led to the differential expression of a large number of genes, with heat shock proteins (HSPs) being the most abundant group between the upregulated genes [16].

Various HSPs are produced in plants under environmental stress conditions such as high temperature, drought, salinity, osmotic, cold and oxidative stresses [17,18,19,20,21,22,23]. It is widely accepted that HSPs play a crucial role in protecting cell structures against stress in plants and other organisms [24,25,26,27,28,29]. Small HSPs (sHSPs) are the most abundant HSPs that are produced universally in prokaryotic and eukaryotic cells upon heat stress [30,31]. In Arabidopsis, sHSP genes are not expressed in unstressed tissues but show high levels of gene expression during heat stress and other environmental challenges [25,32,33]. A number of sHSPs were highly induced on exposure of Arabidopsis plants to heat, osmotic and salinity; with the induction of these genes were more pronounced under a combined treatment of these three stresses [34]. High levels of sHSP gene expression and protein accumulation upon environmental stresses support the hypothesis that these proteins play an important role in stress tolerance [35,36].

For cell survival under stress conditions, the maintenance of proteins in their functional conformations and the prevention of the aggregation of non-native proteins are particularly important. In this regard, HSPs are involved in stabilizing proteins and membranes, and thus they can assist protein refolding under stress conditions [24,37,38,39]. It was reported that sHSPs represent the first line of defense in the cell to prevent protein misfolding [40]. Certain sHSPs, such as HSP18.1 isolated from pea (*Pisum sativum*) and HSP16.6 from *Synechocystis* sp. PCC6803, were shown to bind to unfolded proteins in vitro and this enables further refolding by HSP70/HSP100 complexes [41]. In addition to their chaperone function, it is also suggested that sHSPs modulate fluidity and composition of cell membranes [42].

sHSPs have roles in protection against oxidative stress. For example, overexpression of chloroplastic sHSPs in tomato (*HSP21*) and tobacco (*HSP26)* provided evidence that these sHSP protect photosystem II from oxidative stress [43,44]. The overexpression of an sHSP (*LimHSP16.45*) from the David lily (*Lilium davidii*) in Arabidopsis led to enhanced cell viability under high temperatures, salinity, and oxidative stress [45]. In addition, *LimHSP16.45* overexpressing plants showed greater activity of superoxide dismutase and catalase than control plants, suggesting that LimHSP16.45 can protect plants against abiotic stresses by enhancing enzymes that scavenge ROS, in addition to their roles in preventing irreversible protein aggregation [45].

In previous work, we found that the expression level of the Arabidopsis sHSP gene, *HSP17.4CI*, was highly induced by heat, salt and drought [34,46]. This finding attracted our attention to further study the dynamic changes in expression patterns of this gene under a wide range of stresses. We were particularly interested in finding out the specific patterns of *HSP17.4CI* expression that would shed light on the modes by which different environmental challenges modulate gene expression. Here, we investigated the expression patterns of *HSP17.4CI* under various abiotic, biotic and oxidative stresses, as well as after treatment with stress-related phytohormones. The expression of *HSP17.4CI* was significantly enhanced by stresses that involve ROS production such as heat, cold, salt, drought, high light, the biotrophic pathogen *Pseudomonas syringae,* in addition to phytohormones such as abscisic acid (ABA) and salicylic acid (SA) that are involved in mediating signals of these stresses. Furthermore, by using mutant plants that are deficient in catalase 2 activity and consequently accumulate endogenous H_2_O_2_, we have supported the proposal that the induction of *HSP17.4CI* is modulated by ROS, where its expression was significantly induced in these *cat2* loss-of-function mutants. 

## 2. Results

### 2.1. The Expression of HSP17.4CI is Induced by Various Abiotic Stresses 

**Figure 1:**
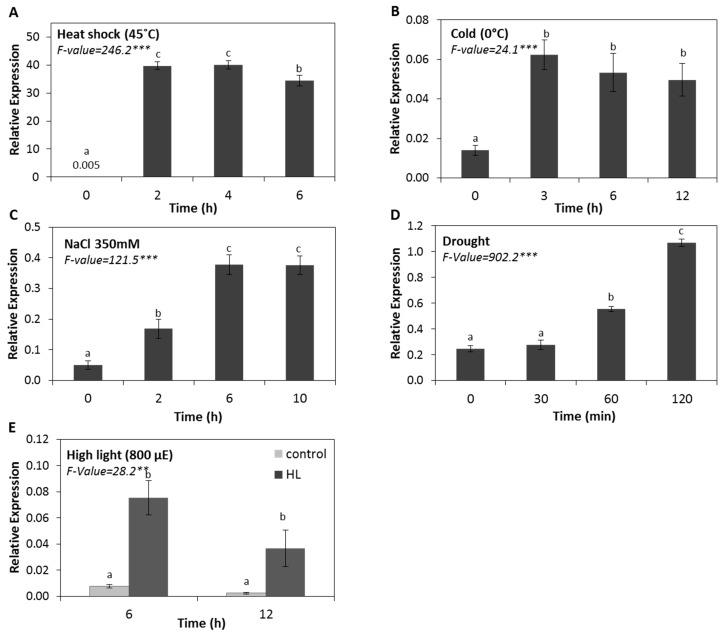
Effect of various abiotic stress treatments on *HSP17.4CI* expression in Arabidopsis. (**A**) heat (45 °C), (**B**) cold (0 °C), (**C**) salt (350 mM), (**D**) drought (plants kept on dry filter paper), (**E**) high light (800 µmol photons.m^−2^.s^−1^). Data represents the average and SDs from three biological replicates. F-values represent one-way ANOVA. **: *p* < 0.01, ***: *p* < 0.001. Means with different letters are significantly different at *p* < 0.05 according to the Tukey HSD test. For panel (**E**), different letters at the same time point indicate significant differences.

To systematically profile the expression pattern of the Arabidopsis *HSP17.4CI*, encoding a cytosolic sHSP, under various stress treatments, we first studied the effect of different abiotic stresses (including heat, cold, salt, drought and high light stress treatments) on the expression pattern of the At*HSP17.4CI* gene. Various growth media used here and sampling time points for each treatment were selected based on previous work [e.g., 46, and references therein]. Our results show that exposure to a heat dose of 45 °C for 2 h, dramatically induced *HSP17.4CI* transcript levels (Figure 1A). The effect of prolonged heat shock at 45 °C for 4 or 6 h on *HSP17.4CI* expression was similar to that observed at 2 h. These results show that *HSP17.4CI* has very low basal expression levels in unstressed plants, but is strongly induced by heat stress. This finding is consistent with previous reports indicating that under normal growth conditions, transcripts of most sHSPs cannot be detected in vegetative tissues, but are rapidly produced in response to heat [35]. The present results also show that cold treatment at 0 °C for 3 h upregulated *HSP17.4CI* expression by more than 4-fold compared to control, with no additional significant increase in the expression level after prolonged cold exposures of 6 or 12 h (Figure 1B). Under salt stress, *HSP17.4CI* was induced by more than 3-fold after 2 h of treatment and 7-fold after 6 h. After 10 h of salt treatment no additional increase in *HSP17.4CI* transcript abundance was detected (Figure 1C). 

Results presented in Figure 1D show that drought treatment led to upregulation of *HSP17.4CI* by about 2-fold after 1 h and by more than 4-fold at 2 h after the treatment. However, there was no detectable increase compared to control after shorter period of 30 min. Furthermore, our results show that *HSP17.4CI* was induced by high-light stress (Figure 1E). Compared to the control, transcript levels of *HSP17.4CI* increased 10 times after 6 h, and 15 times after 12 h of high light exposure at 800 µE (Figure 1E). Altogether, these data indicate that the expression level of the *HSP17.4CI* gene is significantly induced by various abiotic stress treatments.

### 2.2. HSP17.4CI is Responsive to Biotrophic but Not to Necrotrophic Pathogens 

**Figure 2:**
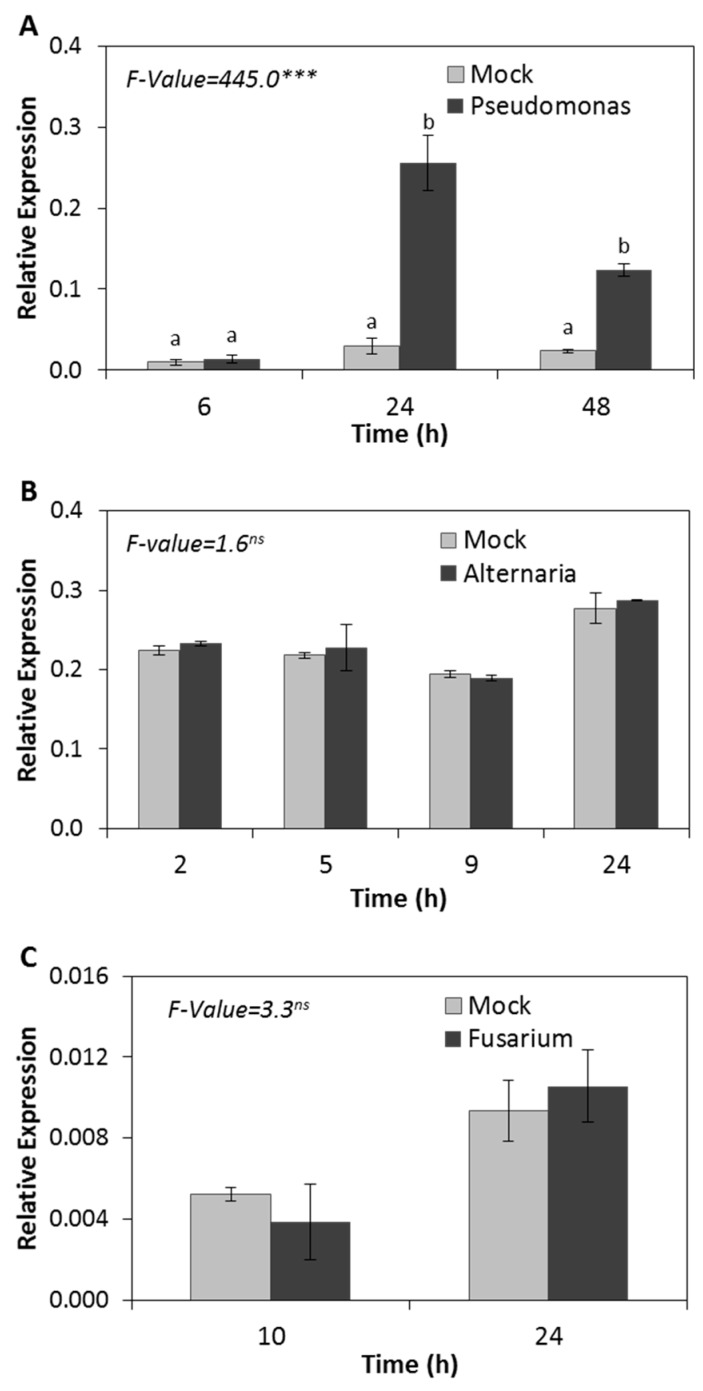
Effect of various biotic stress treatments on *HSP17.4CI* expression in Arabidopsis. (**A**) *Pseudomonas syringae*, (**B**) *Alternaria brassicicola*, (**C**) *Fusarium oxysporum*. Shown are average values and SDs of three biological replicates. F-values represent one-way ANOVA. ***: *p* < 0.001, ns: not significant. Means with different letters at each time point are significantly different at *p* < 0.05 according to the Tukey HSD test.

In addition to their roles in plant tolerance to abiotic stresses, HSPs were reported to play a role in plant defense against biotic stresses [47]. Here, we studied the effect of different pathogens on the expression of the Arabidopsis *HSP17.4CI*. Our study included the biotrophic pathogen *Pseudomonas syringae*, and the necrotrophic pathogens *Alternaria brassicicola* and *Fusarium oxysporum.* We observed no changes in the expression levels of *HSP17.4CI* at 6 h after inoculation of Arabidopsis plants with *P. syringae* (Figure 2A). However, after 24 h from inoculation we observed an increase of *HSP17.4CI* expression by more than 8-fold compared to the mock-treated control. After 48 h from inoculation, the expression level of *HSP17.4CI* was lower than that after 24 hours, but still 4-times higher than the corresponding mock treated control, suggesting a relatively early role for *HSP17.4CI* during defense against this biotrophic pathogen. In contrast, no changes in the expression level of *HSP17.4CI* were found after infection with the necrotrophic pathogens *A. brassicicola* or *F. oxysporum* at any of the indicated time points (Figure 2B,C). 

### 2.3. HSP17.4CI is Upregulated by Plant Hormones ABA and SA but Not by MJ

**Figure 3:**
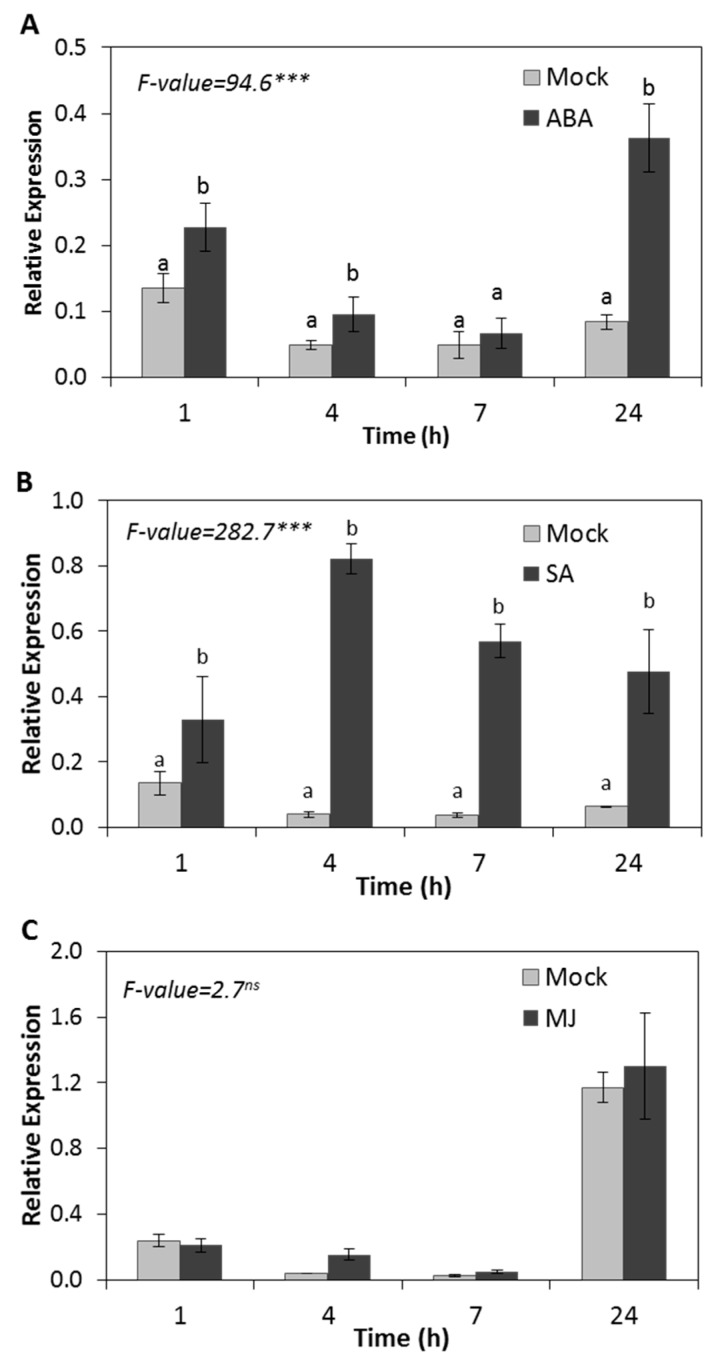
Effect of various plant hormone treatments on *HSP17.4CI* expression in Arabidopsis. (**A**) abscisic acid (ABA, 400 µM), (**B**) salicylic acid (SA, 4 mM), (**C**) methyl jasmonate (MJ, 0.1 μM per liter of air). Data represent the average and SDs of three biological replicates. F-values represent one-way ANOVA. ***: *p* < 0.001, ns: not significant. Means with different letters at each time point are significantly different at *p* < 0.05 according to the Tukey HSD test.

Plant responses to different abiotic and biotic environmental stresses are coordinated by plant hormones such as ABA, SA, and MJ. To investigate the involvement of these plant hormones in the modulation of the expression of *HSP17.4CI*, the transcript level of this gene was monitored in Arabidopsis plants after hormone treatment. Our results show that the *HSP17.4CI* expression level was significantly increased at 1, 4 and 24 h after ABA treatment, reaching more than 4-fold increase after 24 h compared to the control (Figure 3A). Considering the suggestion that ABA regulates plant responses to various abiotic stresses, the induction of *HSP17.4CI* by ABA is in line with *HSP17.4CI* induction by the applied stresses shown in Figure 1. 

The plant hormone SA is known to be involved in plant growth and development and plays a role during defense against biotrophic pathogens [48]. To investigate the possible involvement of SA in the induction of *HSP17.4CI* by the biotrophic pathogen *P. syringe* (Figure 2A), we analyzed *HSP17.4CI* expression in plants treated with SA. Our results show that treating Arabidopsis seedlings with SA significantly induced *HSP17.4CI* at all of the examined time points (Figure 3B). These results are consistent with the induction of this sHSP by the biotrophic pathogen, *P. syringae* (Figure 2A) that activates SA signaling [49]. In contrast to ABA and SA, MJ treatment showed no significant effect on *HSP17.4CI* expression in the treated Arabidopsis plants at any of the investigated time points (Figure 3C). This result is in line with the finding that the expression level of this gene was not affected by the necrotrophic pathogens *A. brassicicola* and *F. oxysporum* (Figure 2B,C), to which the MJ signaling pathway is known to be involved in plant defense [49].

### 2.4. HSP17.4CI is Strongly Upregulated by Oxidative Stress Treatments

**Figure 4:**
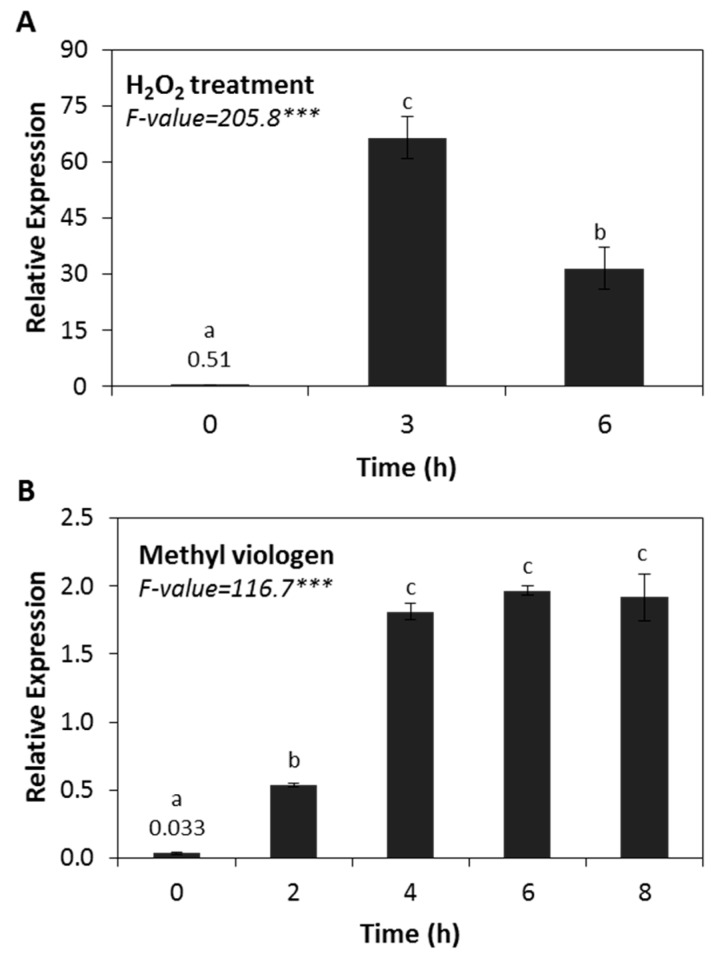
Effect of various oxidative stress treatments on *HSP17.4CI* expression in Arabidopsis. (**A**) H_2_O_2_ treatment (500 mM), (**B**) methyl viologen treatment (30 µM). Shown are average values and SDs of three biological replicates. F-values represent one-way ANOVA. ***: *p* < 0.001. Means with different letters are significantly different at *p* < 0.05 according to the Tukey HSD test.

A general role of sHSPs in oxidative stress tolerance was presumed by observations that production and accumulation of plant sHSPs increase in response to H_2_O_2_ treatments in vitro [35,50]. We found that the expression level of *HSP17.4CI* was strongly induced in Arabidopsis plants treated with H_2_O_2_ (Figure 4A). After only 3 h of H_2_O_2_ treatment, we observed 130-fold higher expression levels than those of the control samples. Similarly, as shown in Figure 4B, transcript levels of *HSP17.4CI* were strongly increased after treatment with methyl viologen, a herbicide that produces superoxide radicals which are converted into H_2_O_2_ by plant superoxide dismutase in chloroplasts. We found that after 2 h of treatment with methyl viologen, the expression level of *HSP17.4CI* was 21-times higher than the untreated control, reaching a maximum level of 71-times higher than the control plants after 4 h (Figure 4B). 

### 2.5. HSP17.4CI Expression is Enhanced in Mutant Plants that Accumulate Endogenous H_2_O_2_

**Figure 5:**
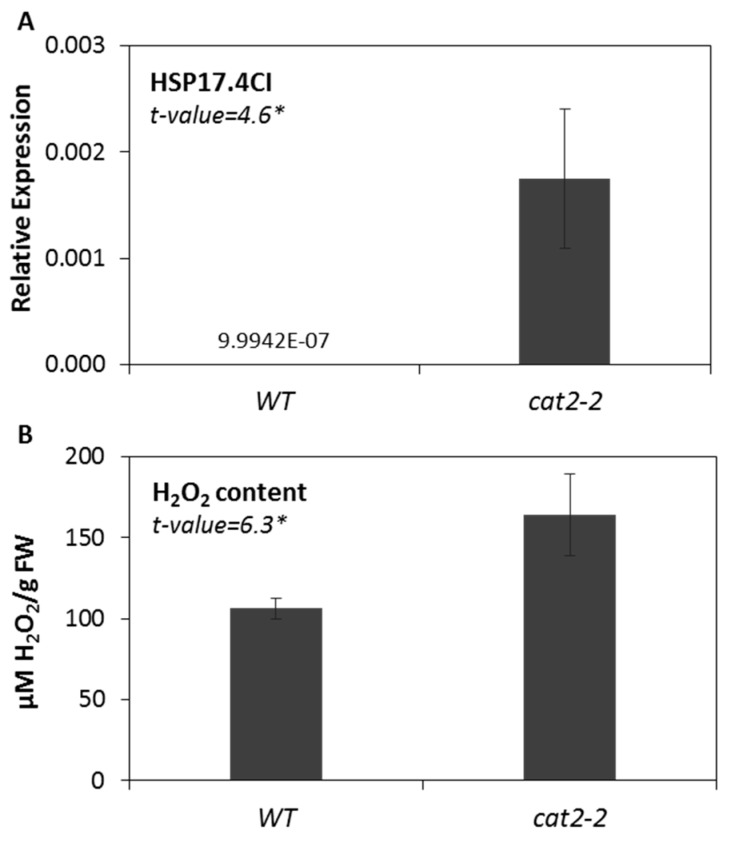
The expression level of *HSP17.4CI* (**A**), and H_2_O_2_ content (**B**) in wild type (WT) and cat2-2 mutant Arabidopsis plants. For *HSP17.4CI* expression experiment, data represents the average and SDs of three biological replicates. For H_2_O_2_ quantification experiment, two biological replicates, with three technical replicates each were used. t-values represent Student’s t-test. *: *p* < 0.05.

To further examine the hypothesis that the observed induction of *HSP17.4CI* under various stress conditions may be mediated by the production of ROS, we used a photorespiratory mutant deficient in catalase 2 activity (*cat2-2*), which accumulate H_2_O_2_ under normal air conditions [6,51]. We found that the expression level of *HSP17.4CI* in the *cat2-2* mutant seedlings was significantly higher than that of the wild type grown in the same conditions (Figure 5A). As expected, we found that the H_2_O_2_ content in *cat2-2* seedlings was significantly higher than that in the wild type (Figure 5B). These data reinforce the hypothesis that the induction of *HSP17.4CI* under various abiotic and biotic stresses would be at least partly mediated by ROS.

### 2.6. Responsiveness of the Arabidopsis sHSPs genes to stress factors

To investigate whether the expression profiles monitored in the current study for the cytosolic sHSP, *HSP17.4CI*, may be shared by other sHSPs, we first cross-examined the Arabidopsis genome for genes annotated as sHSPs [accessed in 34]. A number of 36 genes were found to be annotated as sHSPs or HSP20-like chaperones (Table S1). We considered genome-wide expression data for a number of different stress treatments including heat, drought and salinity [42], as well as oxidative stresses imposed by H_2_O_2_ [52] and methyl viologen [16]. As plants in their natural habitats are usually subjected to combined stresses, and plant response to these combinations cannot be predicted form their responses to individual ones, we also included expression data of *HSP17.4CI* and other sHSPs under a combined treatment of heat, osmotic and salinity [34]. In this study [34], *HSP17.4* was reported to be highly induced by combined stress treatment of heat, drought and salinity. Out of the 36 sHSPs, we found that the expression of 19 genes was differentially expressed by at least on stress treatment (Table 1). Only, *HSP17.6C* showed similar expression pattern to *HSP17.4CI* under the considered abiotic and oxidative stress treatments. With exception of drought stress, another three genes (*HSP17.6A*, *HSP17.6* and *HSP23.5*) were induced by heat, salinity and combined treatments, in addition to oxidative stresses imposed by H_2_O_2_ and methyl viologen (Table 1). Furthermore, another four genes (*HSP17.6B*, *HSP17.6A*, *HSP17.4B* and *HSP15.7*) were induced only by heat and oxidative stresses (Table 1). Notably, two HSP20-like chaperone genes were repressed by multiple (AT4G21870) or heat (AT1G76770) stresses, but not responsive to any of the other treatments.

To find any correlation between subcellular localization of various sHSP genes and their expression patters, we allocated these genes to their subcellular sites (Table 1 and Table S1). Data showed that the sHSP genes that showed similar responsiveness to the expression of *HSP17.4CI* were localized to cytoplasm except *HSP23.5* (mitochondrial) and *HSP15.7* (peroxisomal). 

## 3. Discussion

ROS produced during adaptation to the majority of relatively mild environmental stresses can act as highly controlled signaling molecules with the ability to deliver different environmental cues to the transcriptional machinery [1,9,55]. The induction of HSPs is one important strategy that plants use to protect themselves against different stresses, including oxidative stress [21,24,32,39,56]. In view of the proposed protective role of the Arabidopsis cytosolic class I sHSP, *HSP17CI*, against diverse stress conditions, the aim of the current study was to systematically investigate the expression patterns of *HSP17.4CI* under a wide range of abiotic, biotic and oxidative stress treatments and to investigate whether the expression of *HSP17.4CI* gene under different stress conditions could be linked to the production of ROS. 

Our results showed that after application of various abiotic stresses, including heat, cold, salinity, drought and high light, the expression level of *HSP17.4CI* significantly raised compared to controls (Figure 1). The rapid induction of *HSP17.4CI,* by these treatments suggests that this gene may act in a common protection mechanism against the damaging effects of these stresses. In Arabidopsis, the overexpression of the *Medicago sativa MsHSP17.7* increased the tolerance of the transgenic lines to heat shock, high salinity, oxidative and drought stresses [21]; similarly, the overexpression of two rice sHSPs (*OsSHSP1* and *OsSHSP2*) led to higher germination rates compared to wild-type plants under salt treatment [57]. 

HSPs were reported to be involved in plant defense responses following biotic stress [47,58,59]. Here, we studied the effect of infection with different pathogens on *HSP17.4CI*. Our data show that the inoculation of Arabidopsis plants with *P. syringae* led to an increase of *HSP17.4CI* expression (Figure 2A), suggesting the role of *HSP17.4CI* during defense against this biotrophic pathogen. In contrast, no significant change in the expression level of *HSP17.4CI* was noticed after infection with the necrotrophic pathogens, *A. brassicicola* or *F. oxysporum* (Figure 2B,C). Based on the mode of infection of the biotrophic pathogens that feed on living plant cells and the necrotrophic pathogens that feed on dead cells, plants defend themselves against biotrophic pathogens by rapid induction of an oxidative burst (excessive production of ROS) that leads to hypersensitive response and programmed cell death, while in the case of necrotrophic pathogens, the infected plants respond by avoiding the production of ROS [49]. Thus, the reported upregulation of *HSP17.4CI* by biotrophic but not necrotrophic pathogens suggests that the expression of this sHSP may be at least partially mediated by ROS.

Phytohormones such as ABA, SA, and MJ orchestrate plant responses to different abiotic and biotic environmental stresses [60]. We found that Arabidopsis seedlings treated with ABA have enhanced relative transcription levels of *HSP17.4CI* compared with the untreated control (Figure 3A). Considering the fact that ABA mediates plant responses to various abiotic stresses, especially drought stress, the induction of *HSP17.4CI* expression by ABA is consistent with the induction of expression observed after drought stress treatment (Figure 1D). This suggests that the upregulation of expression of this sHSP by drought could be mediated by ABA signaling, which could integrate a cross-talk with ROS [61,62]. Furthermore, we found that the expression of *HSP17.4CI* rapidly increased after treatment with SA (Figure 3B). These results are in line with the induction of expression of *HSP17.4CI* by infection with the biotrophic pathogen *P. syringae* (Figure 2A), which activates SA signaling [49]. MJ treatment showed no effect on the expression level of *HSP17.4CI* in Arabidopsis seedlings (Figure 3C). This result is consistent with the finding that the expression level of *HSP17.4CI* gene was not affected by *A. brassicicola* (Figure 2B) and *F. oxysporum* (Figure 2C), where MJ signaling pathway is known to be involved in plant defense against these pathogens [49]. The modulation of expression of *HSP17.4CI* observed after treatment with phytohormones, and abiotic and biotic stresses suggests that environmental stresses might influence the expression of *HSP17.4CI* through a cross-talk between hormonal and ROS signaling.

Indeed, almost all environmental stresses enhance ROS production [63,64]. The participation of ROS generated during the applied abiotic and biotic stresses in the induction of *HSP17.4CI* (Figure 1 and Figure 2) is supported by the finding that treatment with cellular oxidants, such as H_2_O_2_ and methyl viologen, also enhanced the expression level of *HSP17.4CI* (Figure 4). These results are in line with the knowledge that expression of various plant sHSPs is increased in response to externally applied oxidative stress [16,35]. Moreover, our results showed that expression of *HSP17.4CI* is also enhanced in catalase loss-of-function plants which accumulate metabolically-produced H_2_O_2_ after their transfer from non-photorespiratory to photorespiratory conditions (Figure 5). 

Reviewing the expression profiles of sHSPs in Arabidopsis genome indicated that only one cytosolic sHSP (*HSP17.6C*) showed identical expression pattern to *HSP17.4CI* under different stress treatments (Table 1). Both genes were reported to be induced by heat, drought, salinity and their combination [42], as well as by oxidative stresses imposed by H_2_O_2_ [52] or methyl viologen [16]. Another three sHSP genes (*HSP17.6A*, *HSP17.6* and *HSP23.5*) showed relatively similar expression behaviors under abiotic and oxidative stress treatments (Table 1).

**Figure 6:**
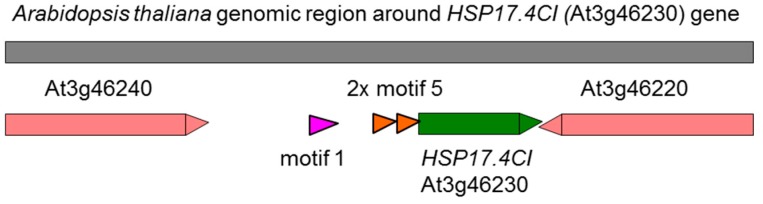
Schematic representation of a 4kb long genomic region surrounding At3g46230 (*HSP17.4CI*, green). Genes are represented as boxes with indicated gene orientation. The identified oxidative stress motifs in the promotor region are highlighted in pink (Motif 1, existed once) and orange (Motif 5, existed twice and indicated as 2x).

Genes that are regulated by oxidative stress share regulatory elements in their promotor sequences that enable concerted changes in gene expression and downstream signaling events [10]. A small number of newly found motifs in promotor sequences is associated with upregulation of the respective genes upon oxidative stress [11,16]. Two of these motifs, Motif 1 ([A/T][A/T]TGGGCCT[T/A]AA) and Motif 5 (GAA[A/C][G/C]TT[C/G][C/A]AGA), are present in the promoter sequence of *HSP17.4CI* (Figure 6). Motif 5 is present in a direct tandem repeat, 22 nucleotide upstream of the 5’ untranslated region (UTR) of *HSP17.4CI*. Motif 1 was found to be more upstream, 219 nucleotide from the start of the 5’UTR. Two members of the heat shock transcription factor family, HSFA2 and HSFA4A, are induced by oxidative stress and suggested to be involved in H_2_O_2_ sensing [10,11]. Interestingly, the finding that Motif 1 is shared by HSFA2 suggest that *HSP17.4CI* may be involved in an oxidative stress signaling pathway that uses a specific subset of HSFs and HSPs to integrate oxidative stress in the flexible gene network that controls the plant’s response to various environmental stress conditions.

In conclusion, the rapid and strong expression of *HSP17.4CI* under a myriad of stress conditions that involve ROS production, suggests that this gene might play an essential role during oxidative stress mitigation. We propose that *HSP17.4CI* can be used as molecular marker for oxidative stress in Arabidopsis and that its orthologs, upon further future studies, could represent candidate target genes for engineering stress tolerant crop plants.

## 4. Materials and Methods 

### 4.1. Plant Materials and Growth Conditions 

*Arabidopsis thaliana* ecotype Columbia (Col-0, wild type) was used in all experiments. Seeds were stratified at 4 °C for 2 days and then transferred to the growth cabinets. For plate-grown Arabidopsis plants, seeds were surface-sterilized and sown on 1x MS (Murashige and Skoog) plates [65]. Plants were grown in cabinets at 24 °C and 15 h photoperiod at 150 µmol photons.m^−2^.s^−1^. Fourteen-day-old Seedlings were exposed for treatments. For soil-grown plants, Arabidopsis seeds were sown on soil (University of California mix) and left to grow in a growth chamber at 24 °C and 8 h photoperiod (150 µmol photons.m^−2^.s^−1^). After around 10 days, seedlings were transplanted to new soil. Four-week-old seedlings were exposed to different treatments. For control samples, plants were mock-treated/inoculated. The treatments were started 1 h after the light turned on to allow direct comparisons between treatments. The growth media and the collection time points for each treatment were chosen based on previous work [e.g., 46, and references therein]. For experiments done on soil grown plants, the above ground plant parts were used for the subsequent analyses. In all measurements, three biological replicates for each treatment were used.

### 4.2. Abiotic Stress Treatments

Heat, cold, salt and water stress treatments were carried out on 14-day-old MS plate-grown seedlings. For heat treatment, plates were moved to an incubator at 45 °C (light intensity was 75 µmol photons.m^−2^.s^−1^). Cold treatment was conducted by incubating the plates on ice in a cold room (temperature was about 0 °C) for the indicated time points. Salt stress was applied by transferring the seedlings from the MS medium to Petri dishes containing water. After 1 h recovery, a NaCl solution was added to make a final concentration of 350 mM. Control samples were left without salt. For drought treatment, seedlings were moved from the MS plates to dry filter paper, whereas control seedlings were moved to distilled-water-wetted filter paper. High light stress was imposed by subjecting 4 week-old soil-grown plants to 800 µmol photons.m^−2^.s^−1^.

### 4.3. Biotic Stress Treatments

All pathogen treatments were performed on 4 week-old soil-grown plants. The pathogens used were *Pseudomonas syringae, Alternaria brassicicola,* and *Fusarium oxysporum.* For the details of *Pseudomonas* inoculations see [46]. *Alternaria* inoculations were carried out as follows: *A. brassicicola* (isolate UQ4273) was grown on agar plates containing V8 vegetable juice (Campbell Soup Co., Camden, NJ). Drops of 5 µL size of a spore suspension (5x10^5^ spores/mL in water) were pipetted onto three to four leaves per plant (one drop per leaf). The plants were then placed in a 20 L-container with a clear polystyrene dome and kept at high humidity. Mock-inoculated control plants were treated the same way with water instead [66]. *Fusarium* inoculations were carried out as follows: *F. oxysporum* was grown on ½ PDB (Potato Dextrose Broth) agar plates at 28°C for one week in the dark. An agar plug was removed from the plate and added to ½ PDB (Potato Dextrose Broth) liquid medium. The inoculum was kept in a shaker for 2 d at 28 °C before draining the culture with a sieve. Using a hemocytometer, the spore number was determined, and then the culture was diluted with distilled water to 10^6^ spores/mL. After carefully removing plants from soil, roots were rinsed with distilled water then dipped in the *Fusarium* spore solution for about 1 min. The inoculated plants were immediately returned to soil and covered with a transparent plastic cover. For the mock-inoculated control roots were dipped in sterilized water instead.

### 4.4. Plant Hormone Treatments

For all hormone treatments, 4-week-old soil-grown plants were used. Solutions of 400 µM ABA or 4 mM SA in 1% ethanol was used for plant spraying. Control plants were sprayed with 1% ethanol solution. In hormone and mock treatments, the pH was adjusted to 5. MJ treatment was applied by taping a piece of cotton containing 100 µL of a 0.8% MJ solution onto the inside wall of a 20 L-container and wrapped in a transparent plastic bag. For mock treatments a piece of cotton containing 100 µL ethanol was used [66].

### 4.5. Oxidative Stress Treatments

Four-week-old soil-grown plants were sprayed with freshly-prepared solutions of H_2_O_2_ (500 mM) or methyl viologen (30 µM), a herbicide that produces superoxide radicals which are converted into H_2_O_2_ by superoxide dismutase in chloroplasts. Both chemicals were dissolved in water. Control plants were sprayed with water.

### 4.6. Growth Conditions of Cat2-2 Plants

Arabidopsis wild type and *cat2-2* [6] seeds were sown on MS plates as described above. After two days at 0 °C, the plates were transferred to a growth cabinet with high CO_2_ level (3000 ppm), at a light intensity of 80 µmol photons.m^−2^.s^−1^, long day conditions (16 h light/8 h dark) and 22 °C day/18 °C night temperatures. After two weeks of growth under the above-mentioned non-photorespiratory conditions, we induced the metabolic formation of H_2_O_2_ by transferring the plates 2 hours after the light switch on to a growth chamber with normal air (CO_2_ ~380 ppm), while keeping all other parameters unchanged. After 8 hours under photorespiratory conditions plants were collected in liquid N_2_ and stored at −80 °C for subsequent analysis. 

### 4.7. H_2_O_2_ Quantification

The optimized Amplex Red-based quantitation method by [67] was applied for H_2_O_2_ estimation. Harvested seedlings (50 mg FW) were ground in liquid N_2_ and re-suspended in 250 µL 50 mM sodium phosphate buffer. The re-suspended tissue was vortexed for 10 s and shaken continuously at room temperature for 30 min. The samples were centrifuged for 5 min at 12,000 rpm at room temperature, and the supernatant was transferred to new tubes. The collected supernatant was re-centrifuged for additional 2 min. The supernatant was transferred to new tubes and kept on ice until analysis (on same day). H_2_O_2_ quantification was conducted according to the manufacturer protocol of Amplex^®^ Red Hydrogen Peroxide/Peroxidase Assay Kit.

### 4.8. RNA Isolation and cDNA Synthesis

After the indicated times for each treatment, the whole rosettes were collected in liquid nitrogen and stored at −80 °C. After grinding in liquid nitrogen, 70 mg plant tissue was used for RNA extraction using the Promega Kit (SV Total RNA Isolation System, spin protocol). The integrity of RNA was tested by gel electrophoresis and quantified by NanoDrop spectrophotometer (ND-1000). For cDNA synthesis, the same amount of RNA (from 1000 to 2000 ng) was used and reverse transcriptase system (SuperScript III, Invitrogen) was used. 

For primer design, the Primer Express 2.0 software (Applied Biosystems) was used. The primers were selected to amplify segments of cDNA of about 100–150 bp and close to the 3′ prime end of the gene. The primer pair used for *HSP17.4CI* (At3g46230) was as follows; forward primer: TCATGAGGAGGTTTCGGTTGC; reverse primer: CTCTCCTGAACTTTCGGCACC. As an internal control, the following *ACTIN* primer mixture was used: Forward universal *ACTIN* AGTGGTCGTACAACCGGTATTGT, *ACTIN2* (At3g18780) reverse GATGGCATGAGGAAGAGAGAAAC, *ACTIN7* (At5g09810) reverse GAGGAAGAGCATTCCCCTCGTA, and *ACTIN8* (At1g49240) reverse GAGGATAGCATGTGGAACTGAGAA.

### 4.9. Real-time RT-PCR

For quantitative RT-PCR analysis, 6 µL-reactions consisted of 3µL SYBR Green master mix reagent (Applied Biosystems), 2 µL primer mixture (forward and reverse, 1 µM each) and 1 µl cDNA (containing the equivalent of 10 ng RNA) were used. Quantitative RT-PCR was performed with an ABI PRISM 7900 Sequence Detection System (SDS) (Applied Biosystems) using SYBR Green to monitor the real-time synthesis of double-stranded DNA. The following thermal cycles were applied; Stage 1: 95 °C 10 min, stage 2: 45 cycles of 95 °C 15 s and 60 °C 1 min, stage 3: one cycle of 95°C 2 min, 60°C 15 s, 95°C 15 s.

SDS 2.2.2 software (Applied Biosystems) was used to produce amplification plots and dissociation curves of the PCR results. For all amplification plots the Rn (normalized reporter signal) was set to 0.2 to get the threshold cycle (C_t_) values. Linear regression analysis (LinReg PCR software) has been applied to calculate the primer efficiency (E value) of each primer pair. The average of primer efficiency for each primer pair in all samples was calculated after excluding the primer efficiencies with R square less than 0.998. The relative expression of each gene (compared to *ACTIN*) was calculated using the following equation: E_S_^^(-CtS)^/E_A_^^(-CtA)^, where E_S_ is the average primer efficiency of the primer pair for this gene, E_A_ is that for *ACTIN*, C_tS_ is the threshold cycle for the studied gene and C_tA_ is that for *ACTIN* [68]. Standard deviation has been calculated from three replicates of each treatment. 

### 4.10. Statistical Analysis

Before performing analysis of variance (ANOVA), the data was tested for its normality of distribution and homogeneity of variance, and whether log-transformation was necessary. The significance of variation in the expression data under various treatments was assessed using one-way ANOVA. The significant differences between the means were identified using Tukey´s HSD test at *p* < 0.05. For *HSP17.4CI* expression in *cat2-2* plants and H_2_O_2_ quantification experiments, the Student’s t-test was used to evaluate the difference between genotypes (wild type (WT) and *cat2-2*). All statistical analyses were performed using SPSS 15.0 software 9 (SPSS Inc., **2006**, Chicago, IL, USA).

## Figures and Tables

**Table 1:** List of Arabidopsis genes annotated as small heat shock proteins (sHSPs) or HSP20-like chaperones and showed expression responsiveness to stress treatments.

Gene Locus	Gene Name	Expression Levels (Log FC) under Stress Treatments						Localization
		Heat	Drought	Salt	Multiple	H_2_O_2_	MV	
AT3G46230	*HSP17.4CI*	6.12	1.50	2.00	7.16	3.52	4.28	Cytoplasm
AT1G53540	*HSP17.6C*	9.08	1.50	3.14	10.18	4.08	3.07	Cytoplasm
AT5G12030	*HSP17.6A*	8.72	-	2.89	9.21	3.70	5.21	Cytoplasm
AT5G12020	*HSP17.6*	7.53	-	1.97	8.22	4.89	4.04	Cytoplasm
AT5G51440	*HSP23.5*	6.34	-	1.50	6.73	3.64	2.02	Mitochondrion
AT2G29500	*HSP17.6B*	5.76	-	-	7.23	4.23	4.48	Cytoplasm
AT1G59860	*HSP17.6A*	4.52	-	-	5.06	4.73	4.63	Cytoplasm
AT1G54050	*HSP17.4B*	2.44	-	-	3.03	3.64	4.08	Cytoplasm
AT5G37670	*HSP15.7*	2.71	-	-	2.95	2.85	1.85	Peroxisome
AT4G10250	*HSP22.0*	8.33	-	-	10.17	1.53	-	ER
AT4G27670	*HSP21*	8.02	-	-	10.04	1.61	-	Chloroplast
AT4G25200	*HSP23.6*	11.23	-	2.73	12.12	-	-	Mitochondrion
AT1G07400	*HSP17.8*	5.96	-	1.50	6.91	-	-	Cytoplasm
AT2G19310	*HSP18.5*	3.47	-	-	3.37	-	-	Cytoplasm
AT1G52560	*HSP26.5*	2.40	-	-	5.12	-	-	Mitochondrion
AT5G59720	*HSP18.2*	1.99	-	-	3.98	-	-	Cytoplasm
AT4G16550	HSP20-like chaperone	1.60	2.17	1.50	1.82	-	-	Unknown
AT4G21870	HSP20-like chaperone	-	-	-	−2.32	-	-	Cytoplasm
AT1G76770	HSP20-like chaperone	−1.79	-	-	-	-	-	Cytoplasm

Data for heat (35 °C, 4 h), drought (imposed by mannitol, 200 mM, 16 h), salt (NaCl, 150 mM, 16 h) and multiple (a combination of the previous three stresses), was adopted from our previous work [42]. For H_2_O_2_ (20 mM, 1 h) and MV (methyl viologen, 50 µM, 2 h), data was quoted from [52] and [16], respectively. The gene names and their subcellular locations were quoted from Uniport [53] and The Arabidopsis Information Resource (TAIR) [54] websites. Expression values with Log FC (fold change) of 1.5 or more compared to controls were considered. All shown values were significantly different at *p* < 0.05. The hyphen “- “means not responsive. ER: endoplasmic reticulum. Genes on the list were ordered where genes induced by both abiotic and oxidative stresses come first, then by the level of induction by heat, with the investigated gene, *HSP17.4CI*, on the top.

## Supplementary Materials

Supplementary materials can be found at https://www.mdpi.com/1422-0067/20/13/3201/s1.
