# Supplementary material for: The AtHSP17.4C1 Gene Expression Is Mediated by Diverse Signals that Link Biotic and Abiotic Stress Factors with ROS and Can Be a Useful Molecular Marker for Oxidative Stress
Source: Int J Mol Sci. 2019 Jun 29;20(13):3201. doi: 10.3390/ijms20133201 (PMC6650836; doi:10.3390/ijms20133201)
Supplement: Supplementary file 1 [file ijms-20-03201-s001.pdf]

Table S1. List of Arabidopsis genes (36 genes) annotated as small heat shock proteins (sHSPs) or HSP20-like chaperones and their expression responses to stress treatments.

Gene locus	Gene name	Expression levels (Log FC) under stress treatments						Localization
		Heat	Drought	Salt	Multiple	H_2O_2	MV	
AT3G46230	HSP17.4CI	6.12	1.50	2.00	7.16	3.52	4.28	Cytoplasm
AT1G53540	HSP17.6C	9.08	1.50	3.14	10.18	4.08	3.07	Cytoplasm
AT5G12030	HSP17.6A	8.72	-	2.89	9.21	3.70	5.21	Cytoplasm
AT5G12020	HSP17.6	7.53	-	1.97	8.22	4.89	4.04	Cytoplasm
AT5G51440	HSP23.5	6.34	-	1.50	6.73	3.64	2.02	Mitochondrion
AT2G29500	HSP17.6B	5.76	-	-	7.23	4.23	4.48	Cytoplasm
AT1G59860	HSP17.6A	4.52	-	-	5.06	4.73	4.63	Cytoplasm
AT1G54050	HSP17.4B	2.44	-	-	3.03	3.64	4.08	Cytoplasm
AT5G37670	HSP15.7	2.71	-	-	2.95	2.85	1.85	Peroxisome
AT4G10250	HSP22.0	8.33	-	-	10.17	1.53	-	ER
AT4G27670	HSP21	8.02	-	-	10.04	1.61	-	Chloroplast
AT4G25200	HSP23.6	11.23	-	2.73	12.12	-	-	Mitochondrion
AT1G07400	HSP17.8	5.96	-	1.50	6.91	-	-	Cytoplasm
AT2G19310	HSP18.5	3.47	-	-	3.37	-	-	Cytoplasm
AT1G52560	HSP26.5	2.40	-	-	5.12	-	-	Mitochondrion
AT5G59720	HSP18.2	1.99	-	-	3.98	-	-	Cytoplasm
AT4G16550	HSP20-like chaperone	1.60	2.17	1.50	1.82	-	-	Unknown
AT4G21870	HSP20-like chaperone	-	-	-	-2.32	-	-	Cytoplasm
AT1G76770	HSP20-like chaperone	-1.79	-	-	-	-	-	Cytoplasm
AT5G54660	HSP21.7	-	-	-	-	-	-	Cytoplasm
AT5G47600	HSP14.7	-	-	-	-	-	-	Cytoplasm
AT2G37570	HSP20-like chaperone, SLT1	-	-	-	-	-	-	Mitochondrion
AT5G53400	HSP20-like chaperone, BOB1	-	-	-	-	-	-	Cytoplasm
AT5G58740	HSP20-like chaperone	-	-	-	-	-	-	Cytoplasm
AT4G27890	HSP20-like chaperone, BOB2	-	-	-	-	-	-	Cytoplasm
AT4G02450	Co-chaperone protein p23-1	-	-	-	-	-	-	Cytoplasm, nucleus, plasma membrane, plasmodesma

AT1G54400	HSP20-like chaperone	-	-	-	-	-	-	Cytoplasm, integral component of membrane
AT5G20970	HSP20-like chaperone	-	-	-	-	-	-	Cytoplasm, integral component of membrane
AT2G27140	Heat shock family protein	-	-	-	-	-	-	Cytoplasm, integral component of membrane
AT3G10680	HSP20-like chaperone	-	-	-	-	-	-	Cell wall, sieve plate, integral component of membrane
AT3G03773	Co-chaperone protein p23-2	-	-	-	-	-	-	Cytoplasm, nucleus
AT1G54840	HSP20-like chaperone, IDM2	-	-	-	-	-	-	Nucleoplasm, nucleus
AT1G76780	HSP20-like chaperone	-	-	-	-	-	-	Integral component of membrane
AT2G03020	HSP20/alpha crystalline family	-	-	-	-	-	-	Cytoplasm
AT4G16540	HSP20/alpha crystalline family	-	-	-	-	-	-	Chloroplast, cytoplasm
AT5G47590	HSP20/alpha crystalline family	-	-	-	-	-	-	Cytoplasm

Data for heat (35 ℃, 4 h), drought (imposed by mannitol, 200 mM, 16 h), salt (NaCl, 150 mM, 16 h) and multiple (a combination of the previous three stresses), was adopted from our previous work [42], genes that showed Log FC of more than one, considered induced genes. For H_2O_2 (20 mM, 1 h) and MV (methyl viologen, 50 µM, 2 h), data was quoted from [52]. and [16], respectively. Expression values with Log FC (fold change) of 1.5 or more compared to controls were considered. The hyphen "-" means not responsive. The gene names and their subcellular locations were quoted from Uniport (https://www.uniprot.org/) and The Arabidopsis Information Resource (TAIR) (https://www.arabidopsis.org/index.jsp) websites. All shown values were significantly different at $p< 0.05$. Genes on the list were ordered where genes induced by both abiotic and oxidative stresses come first, then by the level of induction by heat, with the investigated gene, *HSP17.4CI*, on the top.
